# Non-Small Cell Lung Cancer from Genomics to Therapeutics: A Framework for Community Practice Integration to Arrive at Personalized Therapy Strategies

**DOI:** 10.3390/jcm9061870

**Published:** 2020-06-15

**Authors:** Swapnil Rajurkar, Isa Mambetsariev, Rebecca Pharaon, Benjamin Leach, TingTing Tan, Prakash Kulkarni, Ravi Salgia

**Affiliations:** Department of Medical Oncology and Therapeutics Research, City of Hope, Duarte, CA 91010, USA; srajurkar@coh.org (S.R.); Imambetsariev@coh.org (I.M.); rpharaon@coh.org (R.P.); bleach@coh.org (B.L.); titan@coh.org (T.T.); pkulkarni@coh.org (P.K.)

**Keywords:** non-small cell lung cancer, driver mutations, testing rates, receptor tyrosine kinases, team medicine

## Abstract

Non-small cell lung cancer (NSCLC) is a heterogeneous disease, and therapeutic management has advanced with the identification of various key oncogenic mutations that promote lung cancer tumorigenesis. Subsequent studies have developed targeted therapies against these oncogenes in the hope of personalizing therapy based on the molecular genomics of the tumor. This review presents approved treatments against actionable mutations in NSCLC as well as promising targets and therapies. We also discuss the current status of molecular testing practices in community oncology sites that would help to direct oncologists in lung cancer decision-making. We propose a collaborative framework between community practice and academic sites that can help improve the utilization of personalized strategies in the community, through incorporation of increased testing rates, virtual molecular tumor boards, vendor-based oncology clinical pathways, and an academic-type singular electronic health record system.

## 1. Introduction

Lung cancer remains the leading cause of cancer deaths in the United States and, in 2020, it will be responsible for an estimated 230,000 cases and 135,000 deaths in the US alone [1]. Non-small cell lung cancer (NSCLC) is the major histological subtype that accounts for approximately 85% of all lung cancer cases and encompasses several subtypes, including adenocarcinoma, squamous cell carcinoma, and large cell carcinoma [2]. Despite advances in screening and diagnosis, most patients still present with metastatic disease, at which point surgical intervention is no longer an option [3]. The advent of targeted therapy and immunotherapy has altered the course of treatment for the majority of patients—with molecular testing now a standard recommendation for late-stage lung adenocarcinoma patients. Tyrosine kinase inhibitors (TKIs) that target abnormalities in several genes, such as *ALK* and *EGFR*, have shown better progression-free survival (PFS) as compared with standard chemotherapy in a number of NSCLC trials [4,5,6]. More recently, other molecular markers, including ROS1, RET, NTRK, BRAF, and MET, have delivered similar clinical benefits to patients with late-stage NSCLC [7,8,9,10,11,12]. Furthermore, mature outcome data from second-generation TKIs is showing durable overall survival benefit for patients [13,14], a factor that was previously disputed with earlier TKIs [15].

Several molecular targets that were previously considered “unactionable”, such as KRAS, now have several targeted therapies under consideration with promising early results [16,17]. Nevertheless, for patients without an actionable target or progression of disease, immune checkpoint inhibitors (ICIs) have resulted in durable outcomes and clinical benefit across several NSCLC trials in various lines of therapy [18,19,20,21,22,23,24]. Protein expression testing of programmed death-ligand 1 (PD-L1) has been identified as a potential, though not definitive, biomarker of predicting response to immunotherapy [21,25,26,27]. Beyond tumor response, recent results from KEYNOTE-001 showed that pembrolizumab monotherapy was associated with a 23.2% 5-year overall survival as compared to 15.5% for previously treated patients [28]. However, therapeutic advancements and outcome improvements have not been uniformly applied in practice, with the majority of trials and novel therapies being more prevalent in academic sites as compared to community practice. We previously showed in a retrospective study that in a cohort of 253 patients from nine community practice centers, the molecular testing rate for first-line treatment decisions was 81.75%, with testing for PD-L1 at only 56% [29]. This suggests that while community sites are on pace to improving their testing rates, the current results are inadequate and require more education and understanding of novel upcoming personalized therapies. The purpose of the current review is to shed light on the available and upcoming therapies in lung cancer, to report the gaps in community practice testing rates, and to identify the available tools that can assist in complex lung cancer management and decision-making. 

## 2. Advances in Genomic Testing and Personalized Therapy

In the last 20 years, therapeutic management of lung cancer has progressed from cytotoxic chemotherapies to personalized targeted therapies that act upon specific genomic alterations. Prior to this, while cytotoxic therapies showed a benefit for early-stage disease [30,31], there was no reported outcome benefit in patients with late-stage lung cancer [32]. Following the completion of the multi-billion dollar endeavor of the Human Genome Project in 2003 [33], the development of next-generation sequencing with high-throughput has enabled large-scale parallel sequencing of the lung cancer genome revealing a plethora of genomic targets including EGFR (10–50%), KRAS (25%), ALK (2–7%), ROS1 (1–2%), RET (1%), BRAF (4%), and others [34,35]. Initially, EGFR tyrosine kinase inhibitors were evaluated in unselected populations with mixed responses due to inadequate selection of patients with EGFR alterations [36,37]. However, the results from randomized Phase III trials for EGFR and ALK tyrosine kinase inhibitors [5] led to the acceptance of genomic testing for ALK and EGFR alterations in routine clinical practice, and in turn, led to the development of faster and more efficient next-generation sequencing platforms that were Clinical Laboratory Improvement Amendments (CLIA)-certified and became widely accepted commercially and at academic sites [38]. While first-generation EGFR TKIs, including gefitinib and erlotinib, showed improved progression-free survival, retrospective studies and outcomes data failed to show improvements in overall survival outcomes [13,39,40,41,42]. In contrast to these results, the FLAURA trial for second-generation TKI, osimertinib, showed significant progression-free survival benefit (median PFS 18.9 vs. 10.2 months) and a considerable overall survival benefit of 35.8 months as compared to 27.0 months in the control [43]. The durable survival benefit of targeted therapies had previously been disputed, but recent results from the long-term survival of advanced ALK-rearranged patients treated with crizotinib showed an undisputable benefit of median OS of 6.8 years and a 5-year OS rate of 36% as compared to the historical 2% [44]. Moreover, advances in immunotherapy have yielded similar improvements and KEYNOTE-189 showed that patients who received immunotherapy resulted in a 20% improvement in the overall survival [45].

The promise of precision medicine and the arrival of personalized therapy has transformed lung cancer care with a number of genetic alterations that have come to fruition or are quickly rising with promising trial results, including EGFR, ALK, ROS1, MET, RET, NTRK, BRAF, KRAS, and immunotherapies (Table 1). However, the rapid and dynamic nature of emerging trial results has made lung cancer management difficult and while academic sites are familiar with trial results and the latest available therapies, a community oncologist, who may see a variety of solid tumors, may have difficulty grasping the complexity of these genomic alterations. In our experience at the academic site, actionable alterations were identified in 53.5% of patients with lung cancer, and the use of genomic-informed therapy was associated with improved survival benefit as compared to patients with no actionable alterations [46]. The use of genomic-informed therapy and selective immunotherapy must be standardized within community practice to ensure improved outcomes.

### 2.1. EGFR

The epidermal growth factor receptor is a transmembrane cell-surface receptor that is activated in 10–50% of patients with NSCLC, which varies based on populations and is more common in Asians and nonsmokers [34,48]. The receptors in the EGFR family exist as inactive monomers, but the binding of extracellular growth factors, such as epidermal growth factor (EGF), has been shown to cause receptor dimerization and induced autophosphorylation of the tyrosine kinase domain, with downstream and intercellular signaling cascades that in turn affect cell motility, invasion, proliferation, and angiogenesis [49]. Initial mutations in EGFR were first described in 2004 and activating mutations in EGFR occurring in exons 18–21 of the kinase domain were associated with sensitivity and response to gefitinib and erlotinib [50,51,52]. This led to the selection of patients with adenocarcinoma histology and EGFR alterations and, in 2009, a landmark Phase III Iressa Pan-Asia Study (IPASS) identified clinical responsiveness and increased progression-free survival in EGFR mutant patients who received gefitinib as compared to standard chemotherapy [50]. The landmark Phase III trial, EURTAC, evaluating erlotinib, an EGFR TKI, as a first-line therapy for patients with EGFR mutations, showed an increased median PFS of 9.7 months as compared to 5.2 months with standard chemotherapy [53]. Two other Phase III trials, the OPTIMAL and ENSURE trials, showed a similar improvement with erlotinib and the US Food and Drug Administration (FDA) approved erlotinib as a first-line cancer therapy for EGFR mutation-positive patients [4,53,54]. Similarly, afatinib, a second-generation TKI, received FDA approval in 2013 following two Phase III trials, Lux-Lung 3 and Lux-Lung 6, that both showed improved PFS of 11.1 months and 11 months respectively, as compared to standard chemotherapy in the first-line setting [55,56].

In 2015, efficacy results for patients with exon 19 deletions or exon 21 (L858R) mutations treated with gefitinib showed a 50% objective response rate (ORR) and led to the FDA approval of gefitinib as a first-line therapy for EGFR mutation-positive patients [57]. However, at that time erlotinib became the standard choice of therapy for many EGFR mutated patients, and mechanisms of primary and secondary resistance to TKI therapy began to emerge. The most commonly identified acquired resistance to early-generation TKIs was the T790M substitution, a secondary EGFR mutation in exon 20, that accounted for approximately 60% of cases [53,55,58,59]. The development of mutant selective pyrimidine-based third-generation TKIs that could block the T790M substitution led to the AURA3 trial evaluating osimertinib, a third-generation TKI, as second-line therapy following T790M EGFR TKI resistance [6]. In 2017, the results of the AURA3 trial showed a significantly improved PFS of 10.1 months and a response rate of 71% as compared to standard chemotherapy [6], and this led to the issuance of FDA approval for osimertinib in the second-line setting for EGFR T790M mutation-positive patients treated with first-line EGFR TKI. Compounding results also exhibited higher CNS response rates with osimertinib (40% vs. 17%) and a longer CNS PFS of 11.7 months vs. 5.6 months [60]. Brain metastases occur in approximately 20–40% of EGFR patients at presentation [61,62] and CNS activity of osimertinib hinted at its potential as a first-line therapy. Unsurprisingly, in 2018, the results of the FLAURA trial showed osimertinib as superior in the first-line setting as compared to first-generation TKIs, with a median PFS of 18.9 months (vs. 10.2 months), ORR of 77% (vs. 69%), and a median duration of response (DOR) at 17.6 months (vs. 9.6 months) [13]. This led to the issuance of FDA approval for osimertinib as the first-line therapy option for EGFR mutant lung cancer. Furthermore, mature data from the FLAURA trial also showed a medial overall survival benefit of 38.6 months over 31.8 months in the control and there was a significant improvement in quality of life, a clinical factor that was never previously achieved in first-generation TKIs [43].

However, despite advances in therapy, acquired resistance inevitably occurs, including EGFR-dependent resistance (6–10%), MET and HER2 amplifications (8–17%), small cell lung cancer (SCLC), and squamous cell carcinoma (SCC) transformation (15%), and others [63]. EGFR-dependent resistance includes S768I, L861Q, G719X, and other alterations that are resistant to most first-generation TKIs except for afatinib that was approved for first-line therapy for patients with rare EGFR alterations [64]. Additional TKIs such as poziotinib are currently under consideration for such alterations and Phase II preliminary data showed a response rate of 43% and a median PFS of 5.5 months in previously treated EGFR-mutant patients [65]. Additionally, other TKIs including TAK-788 (NCT03807778), TAS6417 (NCT04036682), and tarloxotinib (TH-4000) (NCT03805841) are currently under investigation in this setting. There are other trials available for less-frequent mutations of EGFR, such as exon 18 or exon 20 EGFR insertions. The availability of numerous EGFR TKIs in the first and refractory setting is strictly contingent upon appropriate assignment to therapy following reflex molecular testing. The improvements in survival are dependent on early identification of molecular markers and appropriate sequence of TKI therapy. In one retrospective study of rates of molecular testing in a community-based academic center, EGFR testing following the approval of reflex testing was only 62% [66]. In another larger cohort of 814 community practice patients, testing rates were similarly low, with only 69% of patients who were tested for EGFR mutations, and approximately 70% of patients who tested positive received appropriate targeted therapy [67]. In a retrospective evaluation of 1,203 advanced NSCLC patients from five community oncology practices, the testing rates of EGFR were at 54% [68]. A comprehensive retrospective cohort of 191 community oncology practices with 5688 patients performed by Flatiron Health, selected patients who were tested for EGFR alterations with either broad genomic sequencing or routine-testing and identified 154 EGFR-mutated patients in the broad-based sequencing group, but reported that only 25% of these patients received appropriate EGFR-targeted therapy [69]. The findings of the study concluded that there was no survival difference between broad-based and routine genomic sequencing, but this misrepresented the utility of broad-based genomic sequencing in the community, as better outcomes cannot be achieved without appropriate assignment to targeted therapy. Meanwhile, in our own community practice experience of 253 patients, we reported testing rates of 94% for EGFR and 96.2% of patients with an EGFR sensitizing mutation received a TKI therapy [29]. The translation of outcomes reported in clinical trials to real-world outcomes requires cooperation and acceptance of molecular testing within community practice and the integration of targeted therapies in community decision-making.

### 2.2. ALK 

ALK, a receptor tyrosine kinase, was originally identified in lung cancer in 2007 with the detection of an echinoderm microtubule-associated protein-like 4 (EML4) gene and anaplastic lymphoma kinase (ALK) gene fusion from a surgically resected lung adenocarcinoma patient [70]. This gene rearrangement is largely independent of EGFR alterations and has been described as an actionable oncogene with incidence in 1–7% of lung cancer patients [71]. ALK-rearranged patients tend to be younger and—similar to EGFR—have a limited history of smoking. Crizotinib, while originally developed as a MET therapeutic, showed a preclinical efficacy for ALK [72]. The Phase I trial lead to the FDA approval of crizotinib in ALK-positive NSCLC [5]. In 2013, the results of the Phase III trial evaluating crizotinib compared to standard chemotherapy showed PFS of 7.7 months (vs. 3.0 months) and ORR of 65% (vs. 20%) [5], resulting in FDA approval of crizotinib for first-line therapy as a standard of care. As with other TKIs, while patients initially respond to ALK inhibitors, resistance invariably develops and one of the most common resistance mechanisms is an acquired ALK mutation (1151Tins, L1152R, C1156Y, F1174V/L, G1269A, and others) [73]. Other resistance mechanisms include EGFR activation, KIT activation, KRAS mutation, and IGF1R activation [74,75,76,77,78,79]. It was estimated that 25% of ALK-mutated patients do not respond to crizotinib in the first-line setting and, in response to these resistance mechanisms [77], other ALK TKIs have been developed. In 2014, the results from the Phase I trial evaluating ceritinib as a potential therapy in ALK-rearranged NSCLC patients with disease progression on crizotinib showed a median progression-free survival of 7.0 months and a response rate of 56% [80]. Based on only the Phase I trial results, the FDA approved ceritinib in patients who have progressed on crizotinib, and in 2017, it expanded its approval for first-line use. Alectinib received similar approval in 2015 in the refractory setting that was later expanded to first-line in 2017 [81,82,83]. In the first-line, alectinib showed a median PFS of 34.8 months with an OS rate of 62.5% as compared to crizotinib with 11 months and 52% [81,82,83]. Brigatinib, a second-generation ALK TKI, was initially identified to have preclinical efficacy and grater potency against all 17 ALK mutants as compared with crizotinib [84,85]. Initial results for brigatinib from a Phase II trial in the refractory setting showed promising responses and yielded FDA approval in 2017 [86]. While alectinib has been shown to be effective against L1196M, C1156Y, and F1174L ALK gatekeeper mutations [87], brigatinib has shown efficacy against ROS1, FLT3, and IGF-1 secondary mutations [88]. The results of the Phase III trial for brigatinib vs. crizotinib in the first-line showed an estimated PFS of 12 months as compared to 11 months with crizotinib, and two-year follow-up data showed brigatinib reduced the risk of progression or death by 76% [14,89]. Several other new generation ALK TKIs including lorlatinib and ensartinib demonstrated 73% and 72% ORR, respectively, following crizotinib and we are awaiting first-line results [90,91].

The availability of a number of ALK inhibitors has complicated management of ALK patients, but in a long-term assessment of 110 patients with an ALK inhibitor, a remarkable OS for advanced ALK NSCLC patients of 6.8 years was reported with 78.4% of patients receiving another ALK inhibitor after first-line progression [44]. Therefore, many studies are reporting that the success of ALK inhibition therapy may lie in the sequence of administrating ALK inhibitors based on metastatic progression and resistance profiles [92,93]. In a retrospective analysis of 31,483 patients with advanced NSCLC at community practices, ALK overall testing rates were 53.1% and rose to 62.1% in 2016, with 21.5% of patients who were initiated into non-targeted therapy before receiving test results [94]. Gierman et al. in 2019 evaluated 1,203 advanced NSCLC patients from five community practices and results showed that only 51% of patients were tested for ALK rearrangement, with approximately 45% of actionable patients receiving targeted therapy [68]. A concurrent study of 814 community practice patients showed that only 65% were tested for ALK alterations [67]. A retrospective study of advanced NSCLC across over 70 community sites in the US showed that only ~50% of patients were tested for ALK alterations during their cancer care [95], suggesting that advancements in liquid biopsies and testing are not translating to real-world practice. The use of liquid biopsies in a large cfDNA study showed that genomic results were concordant with tissue and utilizing cfDNA liquid biopsies increased detection and rates of testing by 48% [96]. The integration of liquid biopsy testing and further controls on tissue biopsy testing may improve the rates of ALK testing and translate the 6.8-year median survival benefit from academic site-wide studies into real-world efficacy. 

### 2.3. ROS1

*ROS1* has been identified as an oncogene in lung cancer and rearrangements have been reported in 1 to 2% of patients with NSCLC [34]. The fusion mutations lead to the dysregulation of the tyrosine-kinase dependent multi-use intracellular signaling pathway, which in turn accelerates growth, proliferation, and progression [97]. Similar to EGFR and ALK alterations, *ROS1* fusions and rearrangements are mutually exclusive and independent of other oncogenes such as KRAS or MET [98]. Following the discovery of *ROS1* fusions in 2007 and in part due to the high degree of homology between *ALK* and *ROS1,* the tyrosine kinase inhibitor crizotinib was explored as a therapeutic option [99,100]. Crizotinib was approved by the FDA in 2016 contingent upon clinical benefit from a PROFILE 1001 Phase I study, where patients had a median PFS of 19.2 months and an ORR of 72% [101]. A Phase II study of ceritinib with 32 patients showed an ORR response rate of 62% and a PFS of 19.3 months for crizotinib-naïve patients, but FDA approval is pending and ceritinib was ineffective against resistance mutations but had activity against CNS disease, as intracranial ORR was 25% and intracranial DCR was 63% [102]. Unlike ceritinib, entrectinib has been shown to be effective against some resistance mutations and had similar CNS activity with a median PFS of 13.6 months and ORR of 55% for patients with CNS disease [103]. This led to the FDA’s approval of entrectinib in the management of ROS1-positive NSCLC. However, lorlatinib is currently the only inhibitor under consideration for *ROS1* that is effective against most resistance mutations and in a Phase II trial it induced an ORR of 26.5% with a PFS of 8.5, with considerable CNS activity inducing an ORR of 52.6% [104]. Other agents such as DS6051b (NCT02279433) and repotrectinib (NCT03093116) are also currently under investigation with results awaiting. A 2018 study by Friends of Cancer Research and Deerfield Institute announced the response of a survey of 157 oncologists and showed that ROS1 testing in the community centers was 32% [105]. However, a comprehensive study of 14,461 patients treated in the community showed testing rates for ROS1 were incrementally lower at 5.7% with 35.5% and 32.9% for *EGFR* and *ALK* respectively [106]. Of the three major approved alterations, ROS1 has the lowest testing rates in several studies [67,105,106]. While tissue biopsies remain the gold standard in detecting *ROS1* fusions and rearrangements, advances in liquid biopsy have shown that it is a viable option for *ROS1* and implementation of this practice may increase the testing rates within the community practice [29,107].

### 2.4. MET 

*MET* oncogenic mutations and amplification has been noted in various solid tumor malignancies, including NSCLC, breast cancer, and head and neck cancer [108,109,110,111,112]. MET alterations or its ligand activation (hepatocyte growth factor) causes the activation of the tyrosine kinase which subsequently activates downstream signaling pathways related to cell growth, apoptosis, motility, and invasiveness [113]. Initially discovered in familial and sporadic papillary renal carcinomas [114], subsequent studies revealed the incidence of *MET* alterations in SCLC and NSCLC, especially MET exon 14 skipping as identified initially by our laboratory [115,116]. *MET* alterations have an incidence rate of 6% in lung adenocarcinoma and 3% of lung squamous cell carcinoma [117,118]. The most frequent alteration is the *MET* exon 14 skipping mutation, which has been identified in 4% of lung cancers. A 2015 study was the first to demonstrate clinical efficacy of crizotinib or cabozantinib in NSCLC patients with *MET* exon 14 skipping mutations [119]. A recent study enrolled 69 NSCLC patients harboring *MET* exon 14 alterations that were treated with crizotinib and reported an ORR of 32% and a median PFS of 7.3 months, suggesting antitumor activity with crizotinib treatment [120]. Several clinical trials, such as the GEOMETRY mono-1 trial and the VISION trial, are evaluating other TKIs like capmatinib and tepotinib in MET exon 14-mutated NSCLC and have shown promising results [12,121]. Interim results of the Phase II GEOMETRY mono-1 trial with 97 enrolled patients reported good ORR and a median PFS of 9.13 months in the treatment-naïve cohort [12]. Recently, capmantinib was granted accelerated FDA approval in metastatic NSCLC patients with *MET* exon 14 skipping mutation, the first TKI approved for MET NSCLC patients. MET amplification, which accounts for 1–4% of NSCLC patients who have not been treated with EGFR TKIs, is associated with a poor prognosis [122,123]. A Phase I trial investigated telisotuzumab vedotin, an antibody-drug conjugate, in NSCLC patients with MET overexpression and demonstrated safety and tolerability of the drug with promising antitumor efficacy [124]. In a study of NGS testing rates of genomic biomarkers in NSCLC patients treated at community sites, only 15% of the 814 patients underwent NGS testing for MET, a sharp decline compared to EGFR (69%) or ALK (65%) testing rates [67]. This testing rate was recapitulated in another community analysis [69], however, MET testing rates were reported as low as 6% in an analysis of NGS screening rates between private clinics, academic centers, and community sites [105].

### 2.5. RET 

Activation of RET results in downstream pathway signaling including MAPK, JAK/STAT, and PI3K/AKT, leading to cell proliferation and migration. Alterations in *RET* are most frequently found in medullary thyroid carcinoma and NSCLC. In NSCLC, RET rearrangements are found in approximately 1–2% of cases [117]. These patients tend to be non- or former light smokers with adenocarcinoma histology and present with advanced disease [125]. Since its discovery, several targeted therapies have been investigated including multikinase inhibitors and selective RET inhibitors. A Phase II trial of RET fusion-positive NSCLC patients were treated with cabozantinib, a TKI targeting RET, VEGFR, and MET. The results demonstrated good clinical efficacy with an ORR of 28% and a median PFS of 5.5 months [126]. The most promising selective RET inhibitors currently under investigation are BLU-677 and selpercatinib (LOXO-292). Interim results from a Phase I clinical trial of 79 RET fusion-positive NSCLC patients treated with BLU-677 demonstrated an ORR of 56% among the 57 evaluable patients and encouraging central nervous system (CNS) activity against brain metastases [127]. The Phase I/II LIBRETTO-001 trial evaluating selpercatinib in a cohort of previously treated NSCLC patients with RET rearrangements (N = 105) also demonstrated marked antitumor efficacy with an ORR of 68%, a remarkable CNS response of 91%, and a median PFS of 18.4 months [8]. In the treatment-naïve cohort (N = 34) of the trial, the ORR was 85%, resulting in the FDA approval of selpercatinib for patients with RET-positive NSCLC. Like MET testing rates, RET demonstrated a 14–15% testing rate in community NSCLC patients [67,69]. Also similar to MET, RET testing rates were reported as low as 8% [105]. This is a staggeringly low rate considering the recent FDA approval and great antitumor activity of selective RET inhibitors.

### 2.6. NTRK 

*NTRK* genes (*NTRK1*, *NTRK2*, and *NTRK3)* encode three TRK proteins (TRKA, TRKB, and TRKC), which play an important role in the cell growth, differentiation, and apoptosis of peripheral and CNS neurons [128]. *NTRK1* and *NTRK2* rearrangements account for 3–4% of NSCLC cases [129]. Several clinical trials have shown the efficacy of TRK inhibitor treatment in *TRK*-positive tumors. Larotrectinib (LOXO-101), a highly selective pan-TRK inhibitor, was first evaluated in a study of 55 pediatric and adult patients with various *TRK* fusion-positive malignancies, four of whom had lung cancer, and reported an ORR of 75% [10]. Remarkably, responses were shown to be durable with a response rate of 71% while 51% of patients stayed progression-free at one year. A multicenter analysis of three major Phase I/II clinical trials—STARTRK-1, STARTRK-2, and ALKA-372-001—investigating entrectinib in 54 patients diagnosed with advanced or metastatic *NTRK*-positive tumors demonstrated an ORR of 57%, a median PFS of 11.2 months, and a median OS of 20.9 months [130]. Larotrectinib and entrectinib are currently FDA-approved for the treatment of advanced *NTRK* fusion-positive NSCLC. Although these clinical trials have shown strong and durable responses to first-generation TRK TKIs, acquired resistance mutations have been identified in colorectal and mammary analogue secretory carcinomas, requiring the development of second-generation TKIs [131,132]. LOXO-195, a second-generation TRK-selective inhibitor, has shown preclinical efficacy and clinical activity in a Phase I trial of *NTRK* fusion-positive cancers previously treated with larotrectinib, demonstrating an ORR of 45% [133,134]. Despite the great clinical response elicited by NTRK-targeted therapies, NTRK testing rates were shown to range from 0–15% in several community site analyses [69,105].

### 2.7. BRAF 

*BRAF* mutations represent 7% of NSCLC cases and are more commonly found in current or former smokers and female patients [117]. The most frequent *BRAF* activating mutation, V600E, carries a poorer prognosis and a shorter disease-free survival [135]. A Phase II trial investigated combination treatments of dabrafenib and trametinib in chemotherapy-pretreated patients diagnosed with *BRAF* V600E-mutated NSCLC and reported an ORR of 63% and a median PFS of 9.7 months in 52 evaluable patients [11]. In a Phase II trial of treatment-naïve patients with *BRAF* V600E-mutated NSCLC, treatment with dabrafenib and trametinib resulted in an ORR of 64% and a median PFS of 10.9 months, although 69% of patients experienced at least one grade 3/4 adverse event [136]. Currently, the combination of dabrafenib and trametinib is FDA approved for the treatment of advanced NSCLC harboring the *BRAF* V600E mutation regardless of the previous therapy. In an analysis by Gutierrez et al., BRAF NGS testing rates in 814 community site patients were reported to be 18%, similar to MET and RET NGS testing rates [67]. Other analyses demonstrated consistent rates of 12–29% [68,69,105]. Interestingly, rates of BRAF testing were shown to be as low as 0.1% in a larger analysis of 14,461 NSCLC patients treated in the community [106].

### 2.8. KRAS 

**Figure 1:**
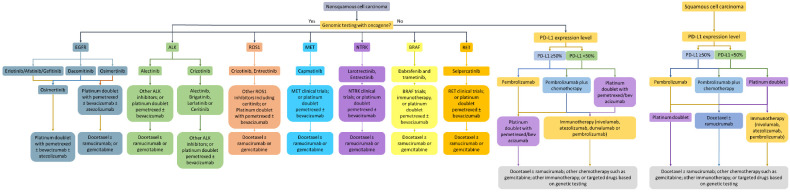
Genomic-informed and immunotherapy-focused management of NSCLC based on approved therapies. The role of immunotherapy is not clear in all of the actionable targets but is currently under investigation.

Alterations in *KRAS*, one of the most frequent oncogenes in solid tumor malignancies, represent up to 32% of lung adenocarcinoma cases [117]. They are generally found in smokers [137] and are associated with a poor prognosis [138], although recent data have reported that it has a minimal effect on overall survival in early-stage NSCLC [139]. Therapeutic targeting of *KRAS* has been notoriously difficult, thus dubbing the molecular marker as an “undruggable” target. However, research into KRAS small molecule inhibitors targeting mutational variants of *KRAS* has shown preclinical and clinical efficacy. AMG-510, an inhibitor targeting KRAS G12C, which accounts for 13% of *KRAS* mutant NSCLC [140], is currently under investigation in a Phase I/II clinical trial of advanced *KRAS* mutant solid tumors. Interim results were recently presented and showed that out of the 29 patients, 10 were diagnosed with NSCLC, of which 90% (*N* = 9) of patients exhibited either a partial response or stable disease [16]. Although there are currently no FDA-approved drugs targeting *KRAS*, small molecule inhibitors like AMG-510 and JNJ-74699157 continue to demonstrate good clinical activity. Another drug, MRTX849, has also shown potent efficacy in vitro and in vivo for G12C positive lung cancer, with pronounced tumor regression in 17 of 26 (65%) KRAS G12C positive cell lines [141]. Preliminary data from the Phase I trial also showed a ~30% decrease in target lesions in heavily pre-treated lung cancer patients [141]. NGS testing of KRAS, although still important now, will become necessary once targeted therapies become approved. In several studies of molecular testing rates in community sites, KRAS testing has widely varied, ranging from 0–43% [66,67,69,105]. As more and more targets such as KRAS become clinically actionable, the landscape of lung cancer therapeutic management will continue to change. However, a number of actionable alterations are currently FDA approved and have distinct therapeutic strategies currently available (Figure 1). 

The testing rates reported in the community have been rising over the years, and the main driver of this transformation has been education and dissemination of novel therapeutics available for the different oncogenes. However, more effort is required as the primary challenge remains that many newly approved targets face an astronomical hurdle in being implemented in daily community practice (Table 2). The most distinct example of this is the testing rates of BRAF reported in community practice at 0.1% in 14,445 patients—the lack of testing also poses a threat towards clinical trial enrollment and delivery of novel therapeutics to patients [106]. 

### 2.9. Immunotherapy

The availability and discovery of more and more targeted therapies makes it a priority that all advanced NSCLC patients are tested at presentation. However, when an actionable alteration is not available, treatment decisions may depend on PD-L1 expression, histology, or the onset of progressive disease. In these situations, immune checkpoint inhibitors have induced response through interaction with cytotoxic T cells, helper T cells, NK cells, macrophages, and other immune mechanisms. In 2015, the first results of monoclonal antibodies against programmed death ligand-1 (PD-1) in the refractory setting showed efficacy of nivolumab, PD-1 inhibitor, with OS (12.2 months) as compared to second-line chemotherapy (9.5 months) [18,19,20]. This led to the FDA approval of nivolumab in advanced NSCLC. Similar approval of pembrolizumab, a PD-1 inhibitor, was contingent upon results from KEYNOTE-001 that showed ORR of 19.4 in refractory NSCLC patients [21]. Soon after, two PD-L1 inhibitors, atezolizumab for stage IV metastatic disease and durvalumab for stage III disease, were also approved based on positive ORRs and OS [22,144]. However, the preliminary analysis reported that PD-L1 expression may be a potential biomarker of response and resistance with only 6.6% of patients whose tumors were negative to PD-L1 responding to durvalumab [22]. In the front-line setting, pembrolizumab was the first immune checkpoint inhibitor (ICI) to demonstrate median PFS of 10.3 months (vs. 6 months) and a response rate of 44.8% (vs. 27.8%) based on the results of KEYNOTE-024 as compared to chemotherapy [145], and it can be utilized as a monotherapy or in combination with chemotherapy depending on PD-L1 expression and the performance status of the patient at presentation [146]. The addition of chemotherapy to pembrolizumab resulted in an increased OS at 12 months of 69.2% (vs. 49.4%) and a median PFS of 8.8 months (vs. 4.9 months), with a comparable adverse event rate of 67.2% vs. 65.8% [147]. These results were surprisingly not recreated when nivolumab was evaluated as a monotherapy, showing a median PFS of 4.2 months with nivolumab vs. 5.9 months, and a similar OS benefit of 14.4 months vs. 13.2 months in the chemotherapy control group [23]. However, it did have success in combination with ipilimumab, showing an improvement in overall survival of 17.1 months vs. 13.9 months with chemotherapy, and a nominal duration of response of 23.3 months (vs. 6.2 months) for the front line setting [148].

Nivolumab plus ipilimumab remains a controversial choice due to grade 3 and 4 adverse events in 32.8% of patients [148]. Atezolizumab monotherapy achieved similar approval with incremental improvements in OS [24], but durvalumab in combination and alone failed to improve survival [149]. While the availability of therapies is beneficial to patients, pembrolizumab is slowly becoming the first-choice option for front-line immunotherapy, partially due to its favorable toxicity profile and versatility as a monotherapy and in combination therapy [150]. However, the availability of therapies has not translated into practice and a retrospective observational study of 55,969 NSCLC patients from the community showed that only 1,344 patients received nivolumab or pembrolizumab in the metastatic setting [142]. More surprisingly, only 8% of these patients were tested for PD-L1 expression [142]. More so, an outcomes study of 423 patients with high PD-L1 who received first-line pembrolizumab monotherapy in the community showed that community clinical outcomes were comparable to clinical trial results with a median PFS of 6.8 months vs. 6.1 months and a median OS of 19.1 months vs. 20 months [151]. A larger study of 10,689 patients in the community showed that utilization of immunotherapy in the first-line is not yet implemented, with <1% of patients treated with immunotherapy in the first-line, but rates were improved in the second and third-line setting [143]. PD-L1 expression was equally underperformed and was tested in <1% of patients [143]. Furthermore, in a quality improvement study of 100 patients who received immunotherapy in the community, only 61% fully completed immunotherapy as planned and 81% had immune-related adverse events [152]. While it is concerning that the reported use of immunotherapy in the community practice is limited, based on experience from melanoma and immunotherapy, the rates are anticipated to slowly increase over time with more education and acceptance of various immunotherapy options [153]. 

While PD-L1 remains an imperfect biomarker, several subgroup analyses in the trials mentioned above show an increased benefit in patients with PD-L1 ≥1% or ≥50%. Therefore, PD-L1 testing should be considered in everyday decision-making, and currently four PD-L1 testing types are available: 22C3, 28-8, SP263, and SP142 [154]. The 22C3 IHC assays were developed alongside pembrolizumab in the Phase I trial as a biomarker for patients who may benefit from treatment [155]. Meanwhile, IHC 28-8 test was developed to be used in conjunction with nivolumab, and SP142 was developed for trial use with atezolizumab [18,19,156,157]. SP263 is the most recent assay that was developed for use with durvalumab, especially in the Stage III setting in NSCLC [156]. All four assays are FDA approved in their individual setting and while testing is not required to initiate treatment, it may support clinical decision-making [156]. Meta-analysis reports show that there is high concordance between 22C3, 28-8, and SP263 assays, but SP142 detected significantly lower PD-L1 expression [154,156]. At the same time, evidence shows that non-commercial laboratory-developed tests (LDTs) used by academic centers detect similar overall percentages of PD-L1 (≥1%) at 63% (vs. 22C3 61%), but PD-L1 ≥50% were much lower at 23% (vs. 22C3 33%) suggesting LDTs are less sensitive than commercial tests [158]. LDTs are becoming more and more utilized in practice and offer a potential solution to the complexity of commercial PD-L1 tests. However, the lack of PD-L1 testing and the difficulty of immune-related toxicities is a challenge that is more difficult to address, and we believe that the integration of community practice with the academic site model is one solution to this grave issue.

## 3. Integration of Personalized Therapy and Molecular Testing in the Community through an Academic Site to Community Practice Network

**Figure 2:**
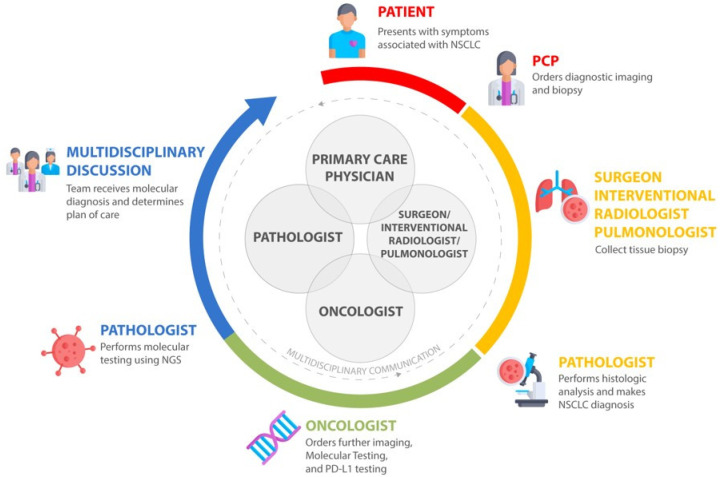
The multidisciplinary care model for community and academic practice integration for lung cancer decision-making.

Advances in targeted therapy and immunotherapy have lowered the costs of molecular testing, making it a viable practice in the academic sites and the community [159]. While academic sites have benefited from a close knowledge of clinical trials and novel therapies, the drive of personalized medicine has not been uniform, with the majority of patients in the community lacking appropriate testing and assignment to therapy [66,67,68,69,94,95,106,142,143,152]. This is especially concerning as the majority of patients or approximately 85% with cancer are treated in the community setting and 50% of collaborative group trial accruals occur in the community [160]. Several models have been proposed to integrate community oncologists into the academic paradigm of personalized medicine, with the most promising being the establishment of interpersonal relationships between community oncologists and academic site physicians through molecular tumor board (MTB) teams [161,162,163,164,165]. The establishment of an MTB team would allow for the proper evaluation of imaging, histopathology, and genomic information that is required to make the appropriate therapeutic decision [166]. One reported study involving 1725 patients who were evaluated through a cloud-based virtual molecular tumor board (VMTB) showed that oncologists chose the VMTB-derived therapies over others, resulting in an increase of matched therapies [165]. Such a model also allows for the dissemination of information regarding available CLIA-certified vendors and platforms for both tissue and liquid biopsy testing that are imperative to improving testing rates and outcomes [167]. The MTB model can be scaled into the community through virtual or physical collaboration, and would further improve collaboration between community sites and academic sites through the interactions between pathologists, oncologists, primary care physicians, radiologists, and pulmonologists in the decision-making process (Figure 2). This team-based approach can be utilized in all cancers, especially during crises such as the recent pandemic of novel coronavirus [168]. The improvement in the relationships with various experts and free-flow of information from the academic site to the community will invariably yield improvements in patient outcomes.

**Figure 3:**
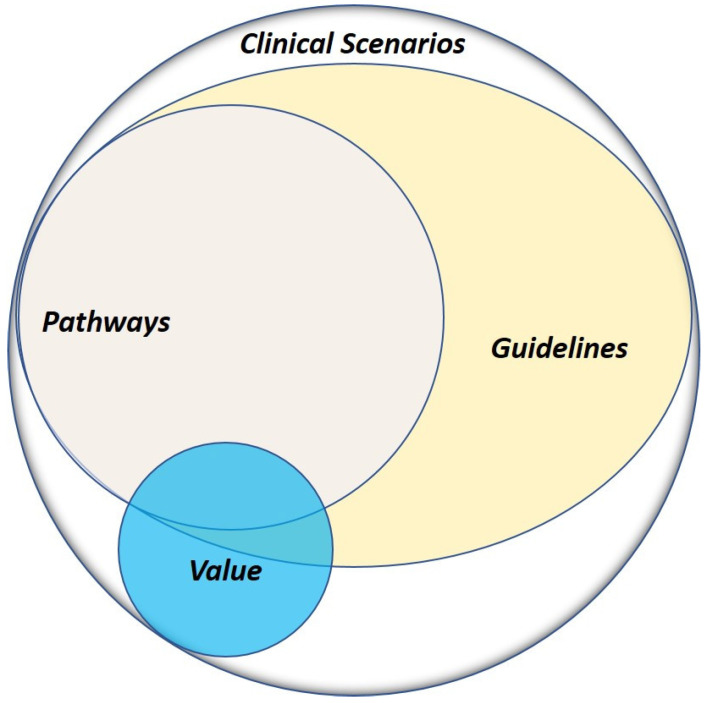
Advantage of guidelines and pathways in clinical scenarios. Patient outcomes are reliant on adherence to evidence-based medicine, which can be facilitated by guidelines and enhanced by pathways.

Another available tool in building the community and academic network is the incorporation of guidelines and pathways into everyday practice. As the majority of oncologists in the community see a number of patients with varying histologies, it is often difficult to keep track of various therapies available, especially for lung cancer. While guidelines such as the National Comprehensive Cancer Network (NCCN) and the American Society of Clinical Oncology provide guidelines regarding the use of immunotherapy and targeted therapy, as well as genomic testing for FDA approved alterations [169], the results in our review show that the gaps in testing rates still remain prevalent and these guidelines are often difficult to interpret during a busy community practice. One proposed solution to this challenge is the implementation of vendor-based oncology clinical pathways (OCPs) that guide physicians in their decision-making based on query questions regarding the patient case [170]. A number of studies have shown that the use of OCPs not only maintains or improves outcomes, but they lower overhead costs for community practice [171,172,173,174]. While guidelines offer multiple recommendations that are difficult to interpret, clinical pathways create a local structure and framework from guidelines or evidence, with the goal of providing the single best therapeutic decision that provides value to the patient (Figure 3) [175]. The advantage of OCPs is not only the availability of decision-making support but the collection of analytics data that can be analyzed for research purposes and continuous quality improvement [176]. An OCP implemented in the community not only evaluates the performance of the community practice, but gives the tools to the community to drive improvements in testing rates and personalized therapy. The wide majority of community practice patients do not consider enrollment in clinical trials, as they are unaware of the option [177]. The pathways incorporate the clinical trials open within the entire enterprise, where trial decisions are placed ahead of other recommendations and always count as on-pathway, which encourages trial enrollment and integrates clinical trials into community practice. Our community practice utilizes the ClinicaPath (formerly ViaOncology) pathway systems in the decision-making process, but there are several vendors available [170].

One recent development in our enterprise is the implementation of a standardized electronic health record (EHR) system in the community that mirrors the academic site medical records in a single system and allows for optimization of testing results and physician referrals for clinical trials. The standardization of molecular testing results and reporting in a fast and reliable manner through the medical record is an important barrier for community oncology practice towards improving testing rates [178]. The cohesiveness of a singular EHR not only results in clinical decision support, but allows the community oncologists to participate in the clinical and translational research process through the evaluation of retrospective patient cohorts in a collaborative model that encompasses a multi-disciplinary team of pathologists, radiologists, and other specialties. The seamless amalgamation of high level genomic and treatment data from the community can be quickly extrapolated from the EHR and utilized in translational studies including evaluation of testing rates and therapy outcomes. This also helps in identifying patients that would be eligible for enrollment in clinical trials available at partnering academic sites, as evidenced by the top accrual rates of the adjuvant EVEREST study in renal cell carcinoma at City of Hope [179]. This is an especially significant strategy to implement in order to enroll and treat older cancer patients who are primarily seen at community sites [180]. Furthermore, the establishment of integrated clinical research has been shown to translate to wider awareness and acceptance of research results, and in 2013, the NCI formed the NCI Community Oncology Research Program (NCORP) [181]. First-cycle results showed that NCORP improved cancer care delivery and access in the community, but challenges remain in growing the program to more organizations across the nation [182]. The evolution of cancer care has to be met with advancements in cancer care and genomic testing access and delivery in community practice. However, the ultimate development of a successful community-based research program requires funding to empower local physicians, infrastructure to support implementation, collaboration between academic and community investigators, and flexibility in operations and organizations.

## 4. Conclusions

The advancements in lung cancer therapy and genomic testing have transformed the lung cancer decision-making process in the last decade. Next-generation sequencing has expanded from a few genes tested with routine testing to broad-based sequencing that has identified a plethora of oncogenes that are involved in driving the progression of NSCLC [183,184,185]. While targeted therapy was initially implemented in the first-line setting, the availability of a number of second- and third-generation TKIs has transitioned from a model of systemic therapy in the refractory setting to a framework of a number of TKIs administered in sequence based on resistance mechanisms and clinical progression of the individual patient [186]. The promise of personalized medicine continues to be realized through the development of ground-breaking immune checkpoint inhibitors and upcoming trials show promise for chimeric antigen receptor (CAR) T-cell therapy [187]. To further realize this mission of precision medicine and to deliver improved outcomes, rigorous clinical data science, and translational research of the care delivery model and access have to be expanded beyond academic sites and into community practice. As we have brought to attention in this review, the community practice, while currently lagging behind academic sites in delivery oncology care, can be systematically and procedurally integrated with academic centers in a unified model for lung cancer decision-making and clinical collaboration. Our identified tools and collaborative concepts, including pathways and MTBs, can be realized in any community setting to enhance communication and trial enrollment. 

## Figures and Tables

**Table 1:** Actionable targets in lung cancer and available therapeutics.

Biomarker Strategy	Approved and Investigational Therapies	Toxicities	Preferred Frontline Therapy	Incidence Rates in NSCLC
EGFR	Osimertinib, Erlotinib, Gefitinib, Afatinib, Dacomitinib	Cutaneous (acneiform rash), gastrointestinal (diarrhea)	Osimertinib	10–50%
ALK	Crizotinib, Ceritinib, Alectinib, Brigatinib, Lorlatinib	Gastrointestinal (nausea, diarrhea), transaminitis, visual changes, pneumonitis	Alectinib	1–7%
ROS1	Crizotinib, Ceritinib, Entrectinib, Lorlatinib	Gastrointestinal (nausea, diarrhea), transaminitis, visual changes, pneumonitis	Crizotinib or Entrectinib	1–2%
MET	Crizotinib, Capmatinib, Tepotinib, Telisotuzumab vedotin	Gastrointestinal, transaminitis	Crizotinib or Capmatinib	3–6%
RET	Cabozantinib, Vandetanib, Sunitinib, Selpercatinib, Pralsetnib(BLU-667) Selpercatinib (LOXO-292)	Fatigue, transaminitis, hypertension, diarrhea	Selpercatinib	1–2%
NTRK	Larotrectinib, Entrectinib, Loxo-195	Fatigue, edema, dizziness, constipation, diarrhea, liver abnormalities	Larotrectinib or Entrectinib	3–4%
BRAF	Dabrafenib, Trametinib, Vemurafenib	Rash, fever, headache, diarrhea	Dabrafenib+Trametinib	7%
PD-L1 expression	Pembrolizumab, Nivolumab, Ipilimumab, Atezolizumab, Durvalumab	Immune-mediated toxicities, including pulmonary and gastrointestinal	Various combination options of chemotherapy and immunotherapy or single-agent immunotherapy	~22–47% [47]

**Table 2:** Reported testing rates of clinically actionable and clinically relevant oncogenes in community practice.

Reported Study	EGFR	ALK	ROS1	MET	RET	NTRK	BRAF	KRAS	PD-L1 Expression
Inal et al. [66]	62%	23%	N/A	N/A	N/A	N/A	N/A	43%	N/A
Gutierrez et al. [67]	69%	65%	25%	15%	14%	N/A	18%	34%	N/A
Gierman et al. [68]	54%	51%	43%	N/A	N/A	N/A	29%	N/A	N/A
Presley et al. [69]	100%	95%	~15%	~15%	~15%	~15%	~15%	~15%	~15%
Illei et al. [94]	N/A	53.1%	N/A	N/A	N/A	N/A	N/A	N/A	N/A
Hussein et al. [95]	~60%	~50%	N/A	N/A	N/A	N/A	N/A	N/A	N/A
Mason et al. [29]	94%	92%	85%	N/A	N/A	N/A	N/A	N/A	56%
Audibert et al. [105]	68%	67%	32%	6%	8%	0%	12%	0%	N/A
Khozin et al. [142]	64%	61%	N/A	N/A	N/A	N/A	N/A	N/A	8.3%
Nadler et al. 2018 [143]	37%	35%	N/A	N/A	N/A	N/A	N/A	N/A	1.2%
Nadler et al. 2019 [106]	35.5%	32.9%	5.7%	N/A	N/A	N/A	0.1%	N/A	5.7%
